# Maternal Exposure to Pyrethroid Insecticides during Pregnancy and Infant Development at 18 Months of Age

**DOI:** 10.3390/ijerph14010052

**Published:** 2017-01-08

**Authors:** Aya Hisada, Jun Yoshinaga, Jie Zhang, Takahiko Katoh, Hiroaki Shiraishi, Kazuhisa Shimodaira, Takashi Okai, Nagako Ariki, Yoko Komine, Miyako Shirakawa, Yumiko Noda, Nobumasa Kato

**Affiliations:** 1 Department of Public Health, Faculty of Life Sciences, Kumamoto University, Honjyo 1-1-1, Chuo-ku, Kumamoto 860-8556, Japan; a_hisada@kumamoto-u.ac.jp (A.H.); katoht@gpo.kumamoto-u.ac.jp (T.K.); 2 Department of Environmental Systems, University of Tokyo, Kashiwanoha 5-1-5, Kashiwa, Chiba 277-8563, Japan; stzhangjie@gmail.com; 3 Faculty of Life Sciences, Toyo University, Izumino 1-1-1, Itakura, Oura, Gunma 374-0193, Japan; 4 National Institute for Environmental Studies, Onogawa 16-2, Tsukuba, Ibaraki 305-8056, Japan; hiroshira@nies.go.jp; 5 Department of Obstetrics and Gynecology, Showa University School of Medicine, Hatanodai 1-5-8, Shinagawa, Tokyo 142-8555, Japan; shimo@med.showa-u.ac.jp (K.S.); okai.t@med.showa-u.ac.jp (T.O.); 6 Department of Psychiatry and Neurology, Showa University School of Medicine, Hatanodai 1-5-8, Shinagawa, Tokyo 142-8555, Japan; nagako.ariki@tyg.jp (N.A.); y-matsu@med.showa-u.ac.jp (Y.K.); miyakoshirakawa@me.com (M.S.); yumiko-be@med.showa-u.ac.jp (Y.N.); katon@med.showa-u.ac.jp (N.K.)

**Keywords:** pyrethroid insecticide, urinary 3-phenoxybenzoic acid, in utero exposure, child development, KIDS

## Abstract

The possible association between maternal exposure to pyrethroid insecticides (PYRs) during pregnancy and infant development was explored. Levels of exposure to PYRs was assessed by metabolite (3-phenoybenzoic acid, 3-PBA) concentration in maternal spot urine sampled in the first trimester of index pregnancy, and infant development was assessed at 18 months of age using the Kinder Infants Development Scale (KIDS), which is based on a questionnaire to the caretaker. The relationship between KIDS score and maternal urinary 3-PBA levels was examined by a stepwise multiple regression analysis using biological attributes of the mother and infant, breast feeding, and nursing environment as covariates. The analysis extracted 3-PBA and the nursing environment as significant to explain the KIDS score at 18 months of age with positive partial regression coefficients. Inclusion of fish consumption frequency of the mother during pregnancy as an independent variable resulted in the selection of fish consumption as significant, while the two variables were marginally insignificant but still with a positive coefficient with the KIDS score. The result suggested a positive effect of maternal PYR exposure on infant development, the reason for which is not clear, but an unknown confounding factor is suspected.

## 1. Introduction

Synthetic pyrethroid insecticides (PYRs) have been widely used for pest control in agricultural, horticultural, forestry, and household settings worldwide. The widespread usage of PYRs in many areas of application gives rise to concern over human exposure to PYRs through ingestion of foods and inhalation as well as dermal contact. In fact, biological monitoring programs revealed frequent detection of PYR metabolites in urine samples from general populations [1,2,3]. 

Although PYRs are considered to have insect-specific toxicity (LD_50_ ratio 4500) [4], it has a known neurotoxicity in mammals at higher doses. Neurobehavioral effects of PYR exposure have been reviewed for developing [5] and adult [6] rodents. The effects in developing animals are particularly relevant because the developing nervous system in fetus and infants would be vulnerable to the toxic action of PYRs, i.e., prolongation of the kinetics of voltage-sensitive sodium ion channels (VSSCs). 

A couple of recent epidemiologic studies explored the possible association between environmentally relevant levels of prenatal PYR exposure and infant neurodevelopment in humans. Berkowitz et al. [7] reported no association between maternal PYR exposure levels and the neonates’ head circumference in 404 mother–neonate pairs in New York City. Horton et al. [8] found no association between permethrin exposure levels of 342 (personal air) or 272 (blood permethrin levels) black and Dominican mothers, respectively, during pregnancy and their infant’s cognitive and motor development at 36 months in New York City. On the other hand, Xue et al. [9] found a significant negative association between PYR exposure levels of 497 Chinese mothers during pregnancy and the neural and mental development of their infants at one year old. Fluegge et al. [10] found significant associations between the concentrations of maternal urinary PYR metabolites and mental neurobehavioral development at 3 months of age in Ohio, USA, though the direction of associations were inverse depending on the urinary metabolite measured for exposure assessment. Other studies explored the possible association between developmental disorders, such as attention-deficit/hyperactivity disorder (ADHD), and prenatal/postnatal pyrethroid exposure and found mixed results [11,12,13].

Taking into consideration the findings of developmental toxicity of PYRs in animal studies [5,14] and ubiquitous exposure among pregnant women [7,15,16], more epidemiologic studies are warranted. The purpose of this study was to explore the possible association between PYR exposure levels of pregnant Japanese women and the development of their infants at 18 months in our prospective cohort.

## 2. Materials and Methods

### 2.1. Subjects

The subjects of this study were 102 pairs each consisting of a mother and her infant. The mothers were recruited to participate in our prospective cohort study when they were in their early pregnancy at a University hospital in Tokyo during 2009–2011 [16,17]. Inclusion criteria were that each mother was (1) living in the Tokyo metropolitan area; (2) 20–50 years of age; and (3) free from any known serious diseases. The mother voluntarily participated in this study after giving informed consent. The ethics committees of University Hospital and University of Tokyo approved this study.

Four hundred and twenty-four mothers were approached and 315 agreed to participate. Blood and urine samples were obtained from 231 of them in the first trimester of gestation (10–12 weeks of gestation). Of the 231, 37 left cohort by the time of delivery for the following reasons: spontaneous abortion (7), change of hospital (12), multiple pregnancies (8), and refusal or other reasons (10). Of these, 147 neonates supplied blood 5 days postpartum [18], and 132 of the mother–baby pairs were followed after delivery until the baby was 18 months of age. All of the data used in the present analysis were obtained from only 102 of the 132 pairs due to missing data for some subjects. Demographic characteristics of the 102 subject mother–infant pairs are shown in Table 1.

### 2.2. Laboratory Analyses

A PYR metabolite, 3-phenoxybenzoic acid (3-PBA), in maternal urine samples obtained at 10–12 weeks of gestation was measured after acidic deconjugation, and solid-phase extraction, and was followed by liquid chromatography-tandem mass spectrometry (Agilent 1100, Agilent Technologies, CA, USA; Micromass Quattro Ultima, Manchester, UK) (LC-MS/MS). Urine volume was corrected by specific gravity of 1.020. Details of the present urinary 3-PBA analysis were given in Zhang et al. [16].

Thyroid stimulating hormone (TSH) concentration in neonatal blood was measured by enzyme-linked immunosorbent assay (ELISA): blood was sampled from the heel of the neonate 5 days postpartum on filter paper and subjected to TSH analysis [18].

### 2.3. Questionnaire Survey of Food Consumption Frequency

Food consumption frequency of the mother was assessed during pregnancy: consumption of “fish”, “shellfish”, “seaweeds”, “meat”, “milk”, “egg”, “soy products”, “vegetable and fruits”, “rice”, “bread”, and “oil” was inquired as to frequency: “none”, “1–2 times a month”, “once a week”, “2–3 times a week”, “everyday”, or “every meal”.

### 2.4. Questionnaire Survey for Development and Nursing Environment

Approximately 1 month before the infant reached 18 months of age, a set of questionnaires was sent to the mother. The set consisted of the Kinder Infant Development Scale (KIDS), Index of Child Care Environment (ICCE), and questionnaire for general information on children, e.g., weight and height, and for breast feeding. 

The KIDS was developed by the Center of Developmental Education and Research, Japan. It consists of a checklist for 9 subscales for children’s behavior: “physical motor”, “manipulation”, “receptive language”, “expressive language”, “language concepts”, “social relationships with children”, “social relationships with adults”, “discipline”, and “feeding”. The check list is rated by caretaker (typically parent) with “yes” or “no”. Based on the answer sheet, the development quotient (DQ) is calculated: the sum of the scores on the 9 subscales was divided by chronological age. It was standardized in 1988 and 1989 using 6000 children aged 0–6 years [19], and recently validated by other scales including Ages & Stages Questionnaire, 3rd edition, family-rated Ability for Basic Movement Scale for Children, Ability for Basic Movement Scale for Children type T, and Functional Independence Measure for Children [20]. 

The ICCE is a parent-rated questionnaire for the practical assessment of children’s home environment. It consists of 13 questions of subscales including “human stimulation”, “social stimulation”, “avoidance of restriction”, and “social support” in a multiple-choice format. The answer is later given a binary score (0/1) according to a manual, and a total score is obtained (0–13). It was validated by a cross-check with Home Observation for Measurements of the Environment (HOME), which is a universally used scale for this purpose [21].

### 2.5. Statistical Analysis

The descriptive statistics were calculated. The Kolmogolov–Smirnov test was applied to see if the distribution of variables was normal, and log-transformation was applied when appropriate. Multiple regression analysis was performed by using a DQ as a dependent variable and urinary 3-PBA concentration as an independent variable. Two models were run. In Model 1, the following covariates were included as independent variables: maternal age at recruitment, maternal body mass index (BMI), gestational week for the index pregnancy, parity (primiparous 0; multiparous 1), infant sex (male 0; female 1), infant body weight at birth, infant blood TSH 5 days postpartum, breast feeding (no 0; yes 1), and ICCE score. Variables potentially relevant to infant development were selected as independent variables. A log-transformed value was used for urinary 3-PBA, maternal BMI, and infant blood TSH. Since the distribution of the ICCE score could not be approximated to normal with any transformation, subjects were dictomized into “high” and “low” by a median ICCE and put into the model. In Model 2, in addition to the independent variables in Model 1, consumption frequency of fish was included. Frequency was dichotomized into frequent eater/less frequent eater. Consumption of fish was included because preliminary analysis of variance found that it was significance <0.1 (*F* = 2.363, *p* = 0.077); however, the number of subjects decreased to 88 because of missing food frequency data from some subjects. It must be noted that maternal smoking during pregnancy was not included as an independent variable in the regression models because no mother smoked. Stepwise variable selection was used to extract the significant variable that explained dependent variable DQ (Pin = 0.05, Pout = 0.1) for both models. Nine subscales of the KIDS were individually used as dependent variables. Distribution of subscale score was not approximated normal by any transformation, each subscale was dichotomized by the median value and discriminant analysis was applied. Independent variables used were the same as multiple regression analysis. Stepwise variable selection was applied. SPSS for Windows ver 19.0 J (IBM Co. Ltd., Tokyo, Japan) was used for statistical analyses. 

## 3. Results

In Table 2, mean and median of urinary 3-PBA concentration (specific gravity adjusted), blood TSH concentration 5 days postpartum, DQ, and ICCE of the 102 subjects (mother or infant) of this study are given. Urinary 3-PBA and blood TSH concentrations of all subjects measured for this cohort have already been reported [18] and the values presented in Table 2 are those of the subpopulation.

Table 3 and Table 4 show the results of stepwise multiple regression analyses of Model 1 and Model 2, respectively. In Model 1, where food consumption was not included in the model, urinary 3-PBA concentration and ICCE were selected as significant, but other variables including maternal age at recruitment, maternal BMI, gestational week, parity, infant sex, infant weight at birth, infant blood TSH concentration 5 days postpartum, infant age at development examination, and breast feeding were not significant for dependent variable DQ (Table 3). Adjusted R^2^ of the model was 0.063. The partial regression coefficient for the two variables were positive, indicating that infants whose mother had higher PYR exposure during pregnancy had a higher DQ score and that infants whose ICCE score was higher had a higher DQ. In Model 2, the significant variable selected was fish consumption frequency alone (Table 4), and urinary 3-PBA and ICCE were marginally insignificant (*p* = 0.070 and 0.079, respectively). Adjusted *R*^2^ = 0.058 and partial regression coefficient for fish consumption was positive (*β* = 0.263), indicating that the DQ of infants was better whose mother consumed fish more frequently during pregnancy.

When discriminant analysis was carried out by using one of the 9 subscales of the KIDS, we divided the subjects into two categories by median score (“higher score” and “lower score”), as dependent variable 3-PBA was selected as significant for “social relationships with children” with a positive canonical correlation coefficient in Model 1 (Table 5), indicating that a higher subscale score was obtained for children whose mother had higher PYR exposure during pregnancy. In Model 2, 3-PBA was not selected as significant for any of the subscales, but fish consumption was selected for “physical motor” and “manipulation” (data not shown).

## 4. Discussion

Median urinary 3-PBA concentration of the present 102 mothers during pregnancy was 0.389 ng/L (Table 2), which was similar to that of the original cohort members (0.361 ng/L, *n* = 231) [16]. As discussed in our previous study [16], the average concentration of 3-PBA in urine samples of the original 231 subjects (and thus that of the present study as well) was similar to that of other Japanese farming/non-farming populations: it was slightly higher in the representative US populations but much lower in Chinese pregnant women.

The result of multiple regression analyses obtained in the present study indicated that maternal PYR exposure during pregnancy was positively associated with infant development at 18 months of age. The significant association with DQ could be ascribed to the association with one of the nine sub-scales: “social relationship with child” (Table 5). The association with DQ was significant in Model 1 (Table 3) and marginally insignificant when fish consumption was included as an independent variable, though the coefficient was close to significance (Table 4). The positive relationship between urinary PYR metabolite concentration and infant DQ was unexpected in light of the previously reported adverse neurobehavioral and neurochemical effects of PYR exposure in fetal and/or developing rodents. PYRs have been known to disrupt normal functions of VSSCs and maintain a prolonged hyperexcitable state. PYRs are also known to induce cholinergic and dopaminergic dysfunctions. These mechanisms are considered to result in increased motor activity, a lack of habituation, and impaired learning and memory in rodents [5,14].

The positive statistical association between the PYR exposure index and infant development obtained in the present study was not consistent with the findings of most of the previous epidemiologic studies: Two studies in the USA and China found a significantly negative association between PYR exposure levels of the mother and infant psychomotor development [9,12]. Another study in the US did not find a significant association between the two [8]. However, Fluegge et al. [10] also found a positive association between the mental neurobehavioral development score (Bayley Scales of Infant Development II, BSID II) of infants at 3 months of age and metabolite (trans-3-(2,2-dichlorovinyl)-2,2 dimethylcyclopropane-1-carboxylic acid, *t*-DCCA) concentration of Type II PYRs in the maternal urine samples collected in the third trimester of gestation. On the other hand, they found a negative association between BSID II score and 3-PBA concentration in maternal urine samples among the same subjects. They ascribed their seemingly contradicting result to different toxicological mechanisms of Type I and Type II PYRs. If the mothers of the present study were mainly exposed to Type II PYRs, e.g., cypermethrin, and not substantially to Type I PYRs, e.g., permethrin, then our result may be in accordance with Fluegge et al.’s result [10]. Analysis of different PYR metabolites is warranted for further interpretation of the present result. 

In the present study, we included frequency of fish consumption as an independent variable for the infant’s DQ in Model 2 multiple regression analysis because the Japanese have a habit of frequent fish consumption, and maternal fish consumption has been known to have a positive effect on children’s development [22]. In fact, we found a significant association between fish consumption frequency of mothers during pregnancy and DQ in a preliminary univariate analysis (ANOVA, *p* < 0.05), and this relationship remained significant in the stepwise multiple regression analysis (Table 4). The beneficial effect of maternal fish consumption during pregnancy for infant development was reproduced in our study. By controlling for fish consumption, the significant association between maternal urinary 3-PBA concentration and ICCE to the infant’s DQ in Model 1 analysis (Table 3) diminished; this may indicate some confounding association between fish consumption and PYR exposure, but bivariate analysis did not find a significant positive association between the two. Even though maternal urinary 3-PBA and ICCE were no longer significant independent variable for DQ after controlling for fish consumption, their positive partial regression coefficients were still close to a significance level; a weak association with DQ, independent of fish consumption, was suggested. It is reasonable to hypothesize that ICCE is positively associated with DQ and some infant subscales; however, the reason for the suggestive positive relationship of PYR exposure index and infant development is not clear.

We found a positive relationship between 3-PBA concentrations in maternal urine sampled during pregnancy and birth weight and head circumference of newborns in this cohort, though the sample size was slightly different from the present study (*n* = 147) [18]. Weight and head circumference at birth has been known to be a predictor of development in infancy and childhood [23,24,25]. Our previous and present findings may be connected.

Since we do not find any supporting evidence that may suggest positive infant psychomotor development by prenatal exposure to PYRs and/or PYR metabolites, this weak positive association between 3-PBA concentration and DQ may rather be inferred as confounding. Higher urinary 3-PBA concentrations were associated with greater vegetable/fruit intake [26,27], which may prevent a delay of mental development by environmental contaminants [28]. Maternal vegetable/fruit intake during pregnancy was also associated with increased weight and the head circumference of offspring at birth [29]. However, it must be mentioned that the frequency of vegetable/fruit consumption of mothers during pregnancy of the present study was not a significant predictor of infant DQ at 18 months (data not shown). It was positively significantly associated with birth weight but independent of maternal urinary 3-PBA [18]. Urinary 3-PBA concentrations were not associated with vegetable/fruit consumption among mothers either. Thus, maternal vegetable/fruit consumption is not likely to be a confounding factor of the association. Another potential factor related to urinary PYR metabolite concentration in the general population was indoor insecticide use [15]; however, it would not be possible to ascribe this to enhanced infant development.

The strength of this study is in the prospective design and the use of biomarkers for exposure assessment. A number of potential covariates were considered for multivariate analysis for the evaluation of infant development. On the other hand, a small sample size might have resulted in a shortage of statistical power to detect weak association(s). The use of parent-rated, simplified assessment scales for infant development (KIDS) and home environment (ICCE) may also be a limitation of this study. Relevant covariates for infant development, i.e., parental economic status and education, were lacking, but they might be reflected in ICCE to some extent. Use of 3-PBA concentration in single spot urine as an indicator of maternal PYR exposure without correction by maternal weight gain during pregnancy may be a limitation of the exposure assessment of this study. Since it is known that PYRs have a short biological half time in the human body, urinary metabolite levels reflect PYR exposure levels over a short period of time before urine sampling; therefore, intra-individual variation in urinary levels is suspected to be large. On the other hand, food habits and household pesticides use, major PYR exposure sources for general population, is individual-specific; therefore, inter-individual variation is also expected to be large. Whether 3-PBA levels in single spot urine is valid as a biomarker of long-term exposure is dependent on the sizes of intra- and inter-individual variations, which should be investigated in a future study. 

## 5. Conclusions

In the present study, we found a suggestive positive association between infant development at 18 months of age and maternal PYR exposure levels during pregnancy, in addition to maternal fish consumption and home environment quality. This weak relationship should rather be inferred as a result of some confounding factor because the beneficial effect of PYRs and/or its metabolites on infant development have not been known; however, no such potential confounding factor(s) was estimated in this study. Further epidemiologic studies with a larger sample size and relevant information on biological and social factors are warranted for a better understanding of the roles of environmental factors in infant development.

## Figures and Tables

**Table 1:** Demographic characteristics of the mother and her infant of the present study (*n* = 102).

Characterititcs	Unit	Mean	Standard Deviation	Median
Maternal age at recruitment	years	34.2	4.8	34.0
Maternal BMI	kg/m^2^	20.6	2.3	20.3
Gestational week	week	38.6	1.4	38.0
Parity of index pregnancy	Primiparous 55; multiparous 47			
Infant sex	Male 45; female 57			
Infant weight at birth	g	2963	376	2959
Infant age at deve-lopment examination	months	18.2	1.3	18.0
Breast feeding	yes 81; no 21			

**Table 2:** Results of outcome variables (*n* = 102).

Outcome Variable	Unit	Mean	Standard Deviation	Median
Urinary 3-PBA #	ng/mL	0.624	0.664	0.389
Blood TSH 5 days postpartum	μIU/mL	2.09	1.74	1.50
DQ *		119	14	122
ICCE **		11.3	1.5	12.0

# Specific gravity adjusted concentration of 3-pehnoxybenzoic acid (3-PBA). * Development quotient of Kinder Infant Development Scale (KIDS). ** Index of Child Care Environment. Full score = 13.

**Table 3:** Result of stepwise multiple regression analysis (Model 1).

Selected Variable	Partial Regression Coefficient		Standardized β	*t*	*p*
	B	95% Confidence interval			
Urinary 3-PBA	3.22	0.58–5.85	0.236	2.423	0.017
ICCE	6.26	0.13–12.38	0.197	2.028	0.045

Dependent variable: Development quotient (DQ). Independent variables not significant were: maternal age at recruitment, maternal body mass index (BMI), gestational week, parity, infant sex, infant weight at birth, infant blood thyroid stimulating hormone (TSH) concentration 5 days postpartum, breast feeding, and infant age at development examination.

**Table 4:** Result of stepwise multiple regression analysis (Model 2).

Selected Variable	Partial Regression Coefficient		Standardized β	*t*	*p*
	B	95% CI			
Fish consumption	7.77	1.65–13.90	0.263	2.524	0.013

Dependent variable: DQ. Independent variables not significant (*p* > 0.1) were: maternal age at recruitment, maternal BMI, gestational week, parity, infant sex, infant weight at birth, infant blood TSH concentration 5 days postpartum, breast feeding, and infant age at development examination. Urinary 3-PBA and ICCE were marginally insignificant (*p* = 0.070 and 0.079, respectively).

**Table 5:** Results of stepwise discriminant analyses * (Model 1).

Dependent Variable (KIDS Subscale)	Independent Variable Selected	Standardized Coefficient of Canonical Discrimination Function	Canonical Correlation Coefficient	Wilks λ	*p*
Physical motor	Maternal BMI	‒**	0.255	0.935	0.010
Expressive language	ICCE	0.664	0.346	0.881	0.006
	Age at exam	0.714			
	Breast feeding	0.600			
Social relationship with child	Urinary 3-PBA	‒**	0.216	0.954	0.030
Feeding	ICCE	0.881	0.367	0.865	0.001
	Parity	0.682			

* Only the results with significant independent variable(s) was selected were shown. Other dependent variables (KIDS subscale) without significant independent variables include “manipulation”, “receptive language”, “language concepts”, “social relationships with adults”, and “discipline”. ** Not available.
